# Prediction of tumor response via a pretreatment MRI radiomics-based nomogram in HCC treated with TACE

**DOI:** 10.1007/s00330-021-07910-0

**Published:** 2021-04-16

**Authors:** Chunli Kong, Zhongwei Zhao, Weiyue Chen, Xiuling Lv, Gaofeng Shu, Miaoqing Ye, Jingjing Song, Xihui Ying, Qiaoyou Weng, Wei Weng, Shiji Fang, Minjiang Chen, Jianfei Tu, Jiansong Ji

**Affiliations:** 1 Key Laboratory of Imaging Diagnosis and Minimally Invasive Intervention Research, Lishui Hospital of Zhejiang University/the Fifth Affiliated Hospital of Wenzhou Medical University, Lishui, 323000 China; 2 Department of Radiology, Affiliated Lishui Hospital of Zhejiang University/the Fifth Affiliated Hospital of Wenzhou Medical University/The Central Hospital of Zhejiang Lishui, Lishui, 323000 China

**Keywords:** Therapeutic chemoembolization, Hepatocellular carcinoma, Prognosis, Nomogram

## Abstract

**Objectives:**

To develop and validate a pre-transcatheter arterial chemoembolization (TACE) MRI-based radiomics model for predicting tumor response in intermediate-advanced hepatocellular carcinoma (HCC) patients.

**Materials:**

Ninety-nine intermediate-advanced HCC patients (69 for training, 30 for validation) treated with TACE were enrolled. MRI examinations were performed before TACE, and the efficacy was evaluated according to the mRECIST criterion 3 months after TACE. A total of 396 radiomics features were extracted from T2-weighted pre-TACE images, and least absolute shrinkage and selection operator (LASSO) regression was applied to feature selection and model construction. The performance of the model was evaluated by receiver operating characteristic (ROC) curves, calibration curves, and decision curves.

**Results:**

The AFP value, Child-Pugh score, and BCLC stage showed a significant difference between the TACE response (TR) and non-TACE response (nTR) patients. Six radiomics features were selected by LASSO and the radiomics score (Rad-score) was calculated as the sum of each feature multiplied by the non-zero coefficient from LASSO. The AUCs of the ROC curve based on Rad-score were 0.812 and 0.866 in the training and validation cohorts, respectively. To improve the diagnostic efficiency, the Rad-score was further integrated with the above clinical indicators to form a novel predictive nomogram. Results suggested that the AUC increased to 0.861 and 0.884 in the training and validation cohorts, respectively. Decision curve analysis showed that the radiomics nomogram was clinically useful.

**Conclusion:**

The radiomics and clinical indicator-based predictive nomogram can well predict TR in intermediate-advanced HCC and can further be applied for auxiliary diagnosis of clinical prognosis.

**Key Points:**

• *The therapeutic outcome of TACE varies greatly even for patients with the same clinicopathologic features*.

• *Radiomics showed excellent performance in predicting the TACE response*.

• *Decision curves demonstrated that the novel predictive model based on the radiomics signature and clinical indicators has great clinical utility*.

**Supplementary Information:**

The online version contains supplementary material available at 10.1007/s00330-021-07910-0.

## Introduction

Hepatocellular carcinoma (HCC) is the most frequent primary malignancy of the liver with high lethality, and its incidence is steadily rising worldwide [1, 2]. Hepatocarcinogenesis is a complex biological process with multiple steps, in which genomic changes induce the formation of cellular intermediates by progressively altering the hepatocellular phenotype, thereby evolving into HCC [3]. Hepatectomy and liver transplantation are considered the only two options with potentially curative effects [4]. Unfortunately, most HCC patients are diagnosed at an intermediate-advanced stage with accompany of extensive diseases or underlying liver dysfunction and are often not eligible for curative therapies. According to the Barcelona Clinic Liver Cancer (BCLC) clinical staging system, local therapy represented by transcatheter arterial chemoembolization (TACE) has become an important therapy for patients with intermediate-advanced liver cancer [5].

As a globally used approach for effective treatment of inoperable HCC, TACE also builds a bridge for connection with other therapies, such as hepatectomy [6] and targeted therapy [7]. However, the therapeutic outcome of TACE varies greatly from patient to patient because the biological behavior of tumor cells is highly heterogeneous [8]. Recent studies have evidenced that the heterogeneity of TACE response also results in a high incidence of local tumor recurrence, which could reach to 46%, 58%, and 63% at 2, 3, and 5 years of posttreatment, respectively [9–11]. Previous studies have confirmed that Cezanne and cytokine signaling 3 methylation are highly related to the heterogeneity of TACE response [8, 12]. Although many efforts have been made, reliable methods related to the prediction of the response to TACE treatment and the prognosis of HCC patients are still limited. To revolutionize the treatment of HCC, an accurate and efficient method for the prediction of TACE response is needed, which is also the key goal of modern personalized medicine [13]. Clinically, magnetic resonance imaging (MRI) plays an essential role in the initial staging, therapeutic strategy, and treatment response assessment of HCC [14]. The current MRI-based diagnosis relies on the experience of radiologists, who make a subjective and qualitative interpretation based on the image. However, it is difficult for them to make a quantitative assessment of tumor heterogeneity [15]. Currently, there is a growing interest in the utilization of radiomics for diagnosis, which could characterize the phenotypes of different diseases by extracting quantitative features from medical images. Previous studies have demonstrated that features based on radiomics are inextricably linked to clinical prognosis and underlying genomic patterns across a range of cancer types [16–18]. Therefore, it may be feasible to predict the TACE response by using radiomics, which could well establish the link between HCC and medical images [19]. To our knowledge, MRI-based radiomics is still inadequate for the prediction of TACE response.

Therefore, the aim of this study was to develop and validate an MRI-based radiomics nomogram that could provide individualized pretreatment evaluations of the TACE response of HCC and to assess the feasibility of its clinical application.

## Materials and methods

### Patient selection

**Fig. 1:**
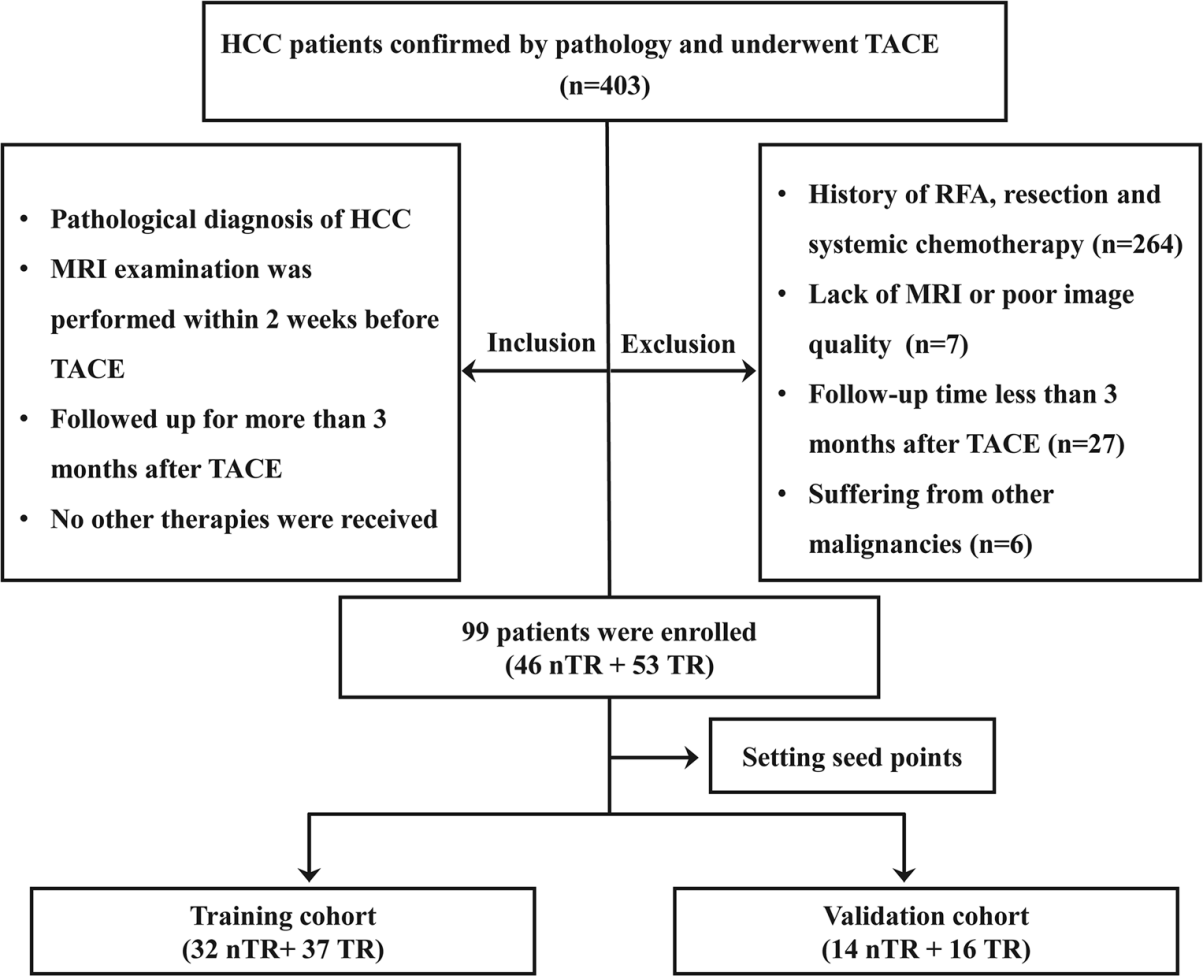
Flowchart of study enrollment

The study was approved by the Institutional Review Board and Human Ethics Committee of Lishui Hospital of Zhejiang University, and the requirement for informed consent was waived. A total of 403 patients who underwent TACE treatment in our institution with pathologically confirmed intermediate (BCLC stage B) or advanced (BCLC stage C) HCC were enrolled from March 2016 to December 2019. Other inclusion criteria for eligible patients included MRI examination performed within 2 weeks before TACE and follow-up for more than 3 months after TACE, as well as no other therapies. A total of 304 patients were excluded due to the following factors: (1) patients who received a combination of other treatments, such as tumor resection, radiotherapy, or systemic chemotherapy (*n* = 264); (2) patients who lacked MR imaging data or poor image quality (*n* = 7); (3) patients with a follow-up time after TACE of less than 3 months (*n* = 27); and (4) patients with other malignancies (*n* = 6). Finally, 99 patients were selected for further study in the present study. The flow of the case identification process is shown in Fig. 1.

### MR imaging protocol

All MRI scans were performed on a Philips ENGENIA 3.0-T MR scanner (Philips Medical Systems). All patients underwent dynamic contrast MRI before and after TACE, and corresponding standard clinical protocols included (1) spectral presaturation with inversion recovery T2-weighted sequence (repetition time/echo time (TE), 3000/200 ms; slice thickness, 7 mm; interslice gap, 1 mm; matrix size, 200 × 195); (2) breath-hold unenhanced and contrast-enhanced (after injection of 0.1 mmol gadopentetate dimeglumine (Guangzhou Kangchen Pharmaceutical Co. Ltd.) per kilogram of body weight) mDIXON-T1WI (water) sequence (repetition time/TE1/TE2, 3.6/1.31/2.2 ms; field of view, 400–314 mm; slice thickness, 5 mm; slice gap, −2.5 mm; matrix size, 224 × 166) were scanned, and 4 dynamic phases were scanned, which were the hepatic arterial phase (15 s), portal venous phase (50 s), substantial period phase (90 s), and delayed phase (180 s); and (3) breath-hold diffusion-weighted echo-planar sequence (field of view, 400–343 mm; matrix size, 116 × 97; repetition time/TE, 2500/64 ms; section thickness, 7 mm; intersection gap, 1 mm; *b* value = 0 and 800 s/mm^2^).

### TACE treatment

All enrolled patients were successfully treated with TACE, including conventional TACE (cTACE) and drug-eluting bead TACE (DEB-TACE). It depends on the physicians to choose cTACE or DEB-TACE based upon the liver function of the patients, the size and number of tumors, whether diffuse or discrete, and their location in the liver. The TACE method recommended by the physician is approved by the patients. All therapeutic procedures were performed according to the current practice guidelines [20] and conducted by interventional radiologists with more than 10 years of clinical experience to ensure the standardization of the treatment process, and successful embolization was determined when no contrast staining in the tumor was identified on postembolization angiography.

For cTACE, lipiodol (Guerbet), gelatin sponge particles, and polyvinyl alcohol were used as embolic agents. The 2.7-Fr microcatheter (Progreat; Terumo) was applied to inject the embolic agents in the hepatic arterial vasculature under the monitoring of digital subtraction angiography (DSA; AlluraClarity FD 20, Philips). All patients were admitted after the cTACE procedures for post-procedure supportive treatment, and routine management was conducted, including hydration, antiemetics, pain control, and monitoring liver function changes.

For DEB-TACE, CalliSpheres® Beads (CB; Jiangsu Hengrui Medicine Co., Ltd.) with a diameter of 100–300 μm were used as carriers and loaded with 60–80 mg epirubicin, pirubicin, or doxorubicin. The treatment process of TACE was similar to that of cTACE, with complete blockage of the tumor-supplying artery as the treatment endpoint.

### Evaluation of TACE response based on the modified Response Evaluation Criteria in Solid Tumors (mRECIST) criterion

**Fig. 2:**
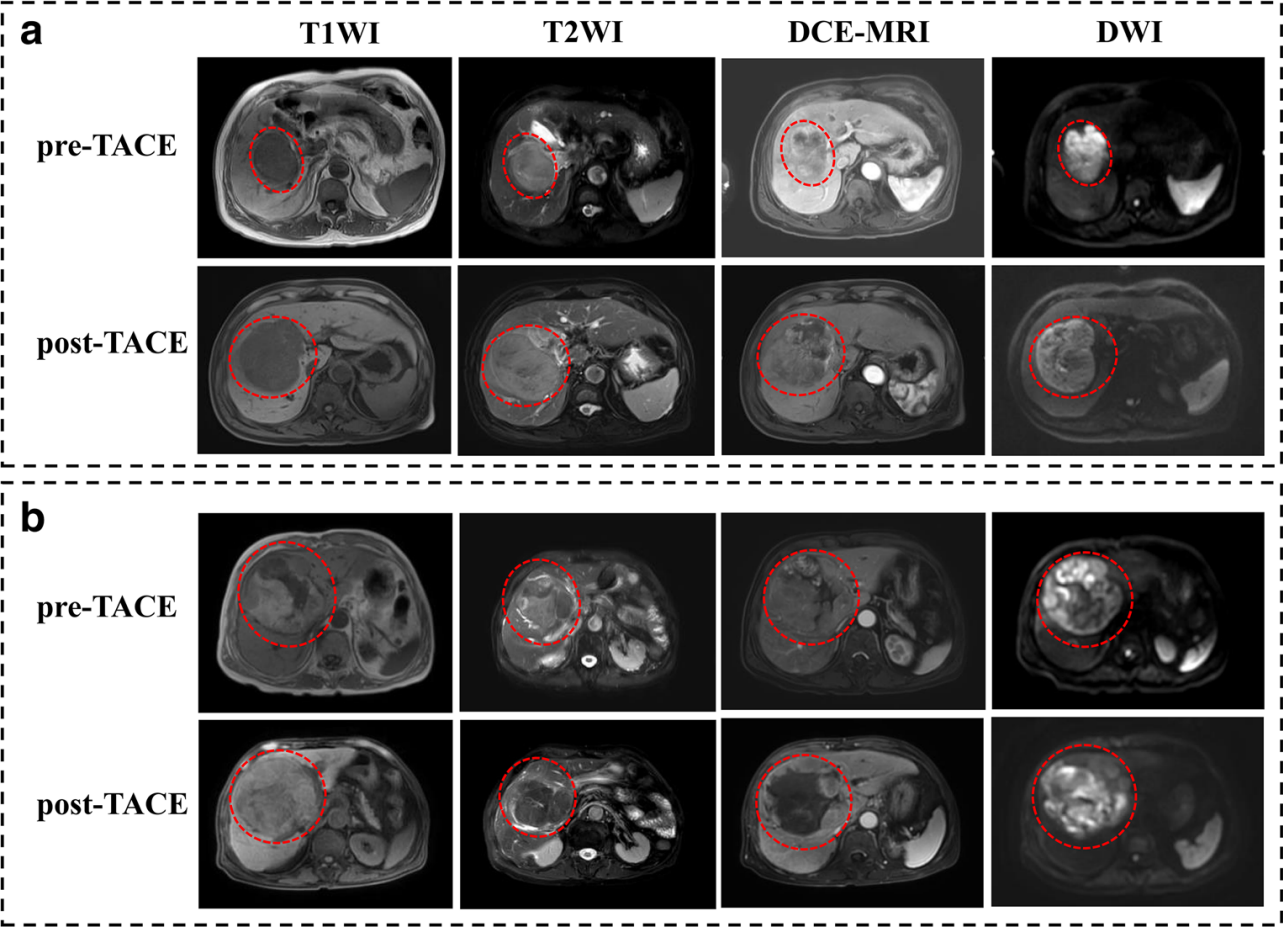
Representative MR images for nTR and TR of HCC patients according to mRECIST criterion. **a** A 73-year-old male HCC patient with a lesion diameter of 69.82 mm underwent MR scanning 1 week before TACE, followed by MR scanning 42 days after TACE. T1WI, T2WI, DCE-MRI, and DWI images were collected, and the results showed that the tumor presented with nTR, with the lesion diameter increasing to 98.79 mm. **b** An 80-year-old male HCC patient, with a lesion diameter of 69.20 mm. MR scanning was performed 1 week before TACE, followed by MR scanning 42 days after TACE, and the results showed the lesion presented with partial response (TR), with the lesion diameter decreasing to 64.32 mm and 85% necrosis. MR, magnetic resonance; TACE, transcatheter arterial chemoembolization; TR, TACE response; nTR, non-TACE response; HCC, hepatocellular carcinoma; mRECIST, modified Response Evaluation Criteria in Solid Tumors

We evaluated the modified Response Evaluation Criteria in Solid Tumors (mRECIST)–based tumor response in patients who underwent TACE treatment by postoperative MRI within 3–4 months. Briefly, the corresponding response of mRECIST [21] included (1) complete response (CR): complete disappearance of the tumor; (2) partial response (PR): a minimum 30% decrease in the sum of diameters of viable target lesions (enhancement in the arterial phase); (3) progressive disease (PD): at least 20% increase in the sum of the diameters of viable (enhancing) target lesions; and (4) stable disease (SD): neither PR nor PD. In the present study, the patients were divided into two groups: the TACE response (TR) group (CR and PR patients) and the non-TACE response (nTR) group (PD and SD patients). Representative MR images for nTR and TR patients are shown in Fig. 2.

The TACE response of patients in this study was determined by two experienced radiologists based on the follow-up MR images. Of the 99 enrolled patients, 46 patients were assigned to the nTR group and 53 patients were assigned to the TR group. Then, the patients were randomly divided into a training cohort (69 patients; nTR = 32, TR = 37) and a validation cohort (30 patients; nTR = 14, TR = 16) by setting seed points.

### Texture feature extraction

All preoperative T2-weighted images were imported into ITK-SNAP (www.itk-snap.org) to delineate the volume of interest (VOI) of tumor areas, which was independently conducted by two radiologists with more than 15 years of experience in clinical diagnosis to secure the reproducibility of the intra-observer and inter-observer segmentation.

Then, the quantitative features of VOIs were calculated by Artificial Intelligence Kit software (A.K. software; GE Healthcare), and a total of 396 radiomics features were extracted for further analysis, which were divided into five categories, including histogram, form factors, gray-level size zone matrix (GLSZM), gray-level co-occurrence matrix (GLCM), and run-length matrix (RLM).

### Selection of radiomics features and construction of the radiomics signature

All extracted radiomics features were further processed for dimension reduction, and the Z-score method was first used to standardize the features before feature dimension reduction, which could remove the unit limits on the data of each feature. Then, the abnormal values were replaced with the median of the parameter in all cases, and the training cohort was used to build the radiomics model, which was further verified by the validation cohort. Feature selection was performed with the least absolute shrinkage and selection operator (LASSO) logistic regression algorithm, and finally, the highly correlated features were screened. Then, the radiomics score (Rad-score) for each patient was calculated by using a linear combination of the cluster of the selected features, which were weighted by their corresponding LASSO coefficients. The predictive accuracy of the radiomics signature was quantified by the area under the receiver operating characteristic curve (AUC) in both the training and validation cohorts.

Furthermore, to improve the prediction performance of the current Rad-score-based model, we further introduced the clinical indicators that were highly correlated with TACE response into our predictive model. Univariate analysis and multivariate analysis were applied to select the highly correlated indicators for each potential clinical indicator, and then combined with the Rad-score to construct a radiomics nomogram as the predictive model for TACE response. The receiver operating characteristic (ROC) curves were plotted to evaluate the predictive ability of the model. Furthermore, to assess the calibration and clinical utility of the nomogram, calibration curves and decision curves were developed. Finally, the model was validated in the validation cohort by using the formula constructed in the training cohort to calculate the AUC and develop the calibration curve.

### Intra-observer and inter-observer agreement

The intra-observer agreement and inter-observer agreement of feature extraction were evaluated by correlation coefficients (ICCs). To compute the intra-observer ICC, 40 T2W MR images were selected randomly and segmented twice in 1 month by reader A. To compute the inter-observer ICC, the selected images were segmented by two radiologists independently (reader A and reader B). Segmentation was performed to further obtain independent feature extraction to compute the intra-observer and inter-observer ICCs. When the ICC was greater than 0.75, it was considered good agreement, and the remaining segmentation was performed by reader A.

### Statistical analysis

All quantitative features were analyzed with R software (version 3.6.1). The differences in clinical indicators between the training and validation cohorts were assessed by using the Kolmogorov-Smirnov test, and Student’s *t* test. The LASSO logistic regression model was performed using the “glmnet” package. The “pROC” package was used to plot ROC curves. The nomogram and calibration curve were depicted using the “rms” package. The correlation analysis between radiomics features was performed using the “pheatmap” package. The clinical utility of our model was evaluated by a decision curve, which was plotted using the “rmda” package [22]. The reported statistical significance levels were all two-sided, with statistical significance set at 0.05.

## Results

### Clinical features of patients and logistic regression analysis of factors related to TR

In this study, a total of 99 patients who received TACE treatment with different responsiveness levels were enrolled according to the inclusion and exclusion criteria. The baseline characteristics of the nTR and TR patients are listed in Table 1. Among them, 50 patients received cTACE treatment, 49 patients received DEB-TACE treatment, and 53 patients had responding, but 46 patients had no responding. Forty males (87.0%) and 6 females (13.0%) were included in the nTR group, with a median age of 54.5 years, and 44 males (83.0%) and 9 females (17.0%) were included in the TR group, with a median age of 65 years. At the initial diagnosis, the median diameter of the tumor was 5.3 cm (range from 3.60 to 9.15 cm) and 5.1 cm (range from 2.825 to 8.175 cm) in the nTR and TR groups, respectively. No significant difference was found in terms of sex, hepatitis B surface antigen (HBsAg), total bilirubin, carcinoembryonic antigen (CEA), tumor diameter, or therapy method between the nTR and TR groups (*p* > 0.05). Meanwhile, there were large differences between the two groups in Child-Pugh class (*p* = 0.009), BCLC stage (*p* = 0.007), and alpha-fetoprotein (AFP) level (*p* = 0.003).

**Table 1:** Baseline characteristics of the patients in the nTR and TR groups

Variables	Univariate analysis			Multivariate analysis	
	nTR (*n* = 46)	TR (*n* = 53)	*p* value	OR (95% CI)	*p* value
Age	54.5 (50, 65.25)	65.0 (50.0, 71.5)	0.054		
Sex			0.586		
Male	40 (87.0%)	44 (83.0%)			
Female	6 (13.0%)	9 (17.0%)			
Child-Pugh			0.009	2.846 (1.054, 7.680)	0.039
A	23 (50.0%)	40 (75.5%)			
B	23 (50.0%)	13 (24.5%)			
BCLC stage			0.007	2.904 (1.136, 7.426)	0.026
B	18 (39.1%)	35 (66.0%)			
C	28 (60.9%)	18 (34.0%)			
HBsAg			0.492		
Positive	36 (81.8%)	38 (76.0%)			
Negative	8 (18.2%)	12 (24.0%)			
AFP (ng/ml)	134.1 (18.9, 2000)	21.7 (5.2, 75.275)	0.003	1.001 (1.000, 1.002)	0.006
TBIL (μmol/L)	19.15 (11.05, 27.475)	15.7 (11.7, 19.7)	0.059		
CEA (U/ml)	2.9 (2.0, 4.25)	3.0 (2.1, 4.5)	0.911		
TD (cm)	5.3 (3.6, 9.15)	5.1 (2.825, 8.175)	0.458		
Therapy method			0.476		
cTACE	25 (54.3%)	25 (47.2%)			
DEB-TACE	21 (45.7%)	28 (52.8%)			

Skewness and kurtosis test was used to test the normality of continuous variables. Independent sample *t* test was used to compare continuous variables with normal distribution. Mann-Whitney *U*-test was used to compare continuous variables with abnormal distribution. Chi-square test was used for the comparisons of categorical variables. *p* value < 0.05 indicates a significant difference in patients’ characteristics between the nTR and TR patients

*TACE*, transcatheter arterial chemoembolization; *nTR*, non-TACE response; *TR*, TACE response; *Child-Pugh*, Child-Pugh-Turcotte score; *BCLC*, Barcelona Clinic Liver Cancer; *HBsAg*, hepatitis B surface antigen; *AFP*, alpha-fetoprotein; *TBIL*, total bilirubin; *CEA*, carcinoembryonic antigen; *TD*, tumor diameter; *cTACE*, conventional TACE; *DEB-TACE*, drug-eluting bead TACE

Multivariate logistic regression analysis revealed that the Child-Pugh class, BCLC stage, and AFP level could be used as predictive factors for TR, as shown in Table 1. The ORs for Child-Pugh class, BCLC stage, and AFP level were 2.846 (95% CI: 1.054–7.680; *p* = 0.039), 2.904 (95% CI: 1.136–7.426; *p* = 0.036), and 1.001 (95% CI: 1.000–1.002; *p* = 0.006), respectively. All the factors remained significant in both univariate analysis and multivariate analysis.

### Intra-observer and inter-observer reproducibility of radiomics feature extraction

There was no statistically significant difference between the measurements of the two readers for each selected feature, with *p* values ranging from 0.712 to 0.861. The intra-observer ICC calculated based on two measurements obtained by reader A ranged from 0.821 to 0.946. The inter-observer agreement between the two readers ranged from 0.767 to 0.910. The results indicated favorable intra- and inter-observer feature extraction reproducibility. Finally, all outcomes were based on the measurement of reader A.

### Radiomics model based on the Rad-score and clinical predictive factors

**Fig. 3:**
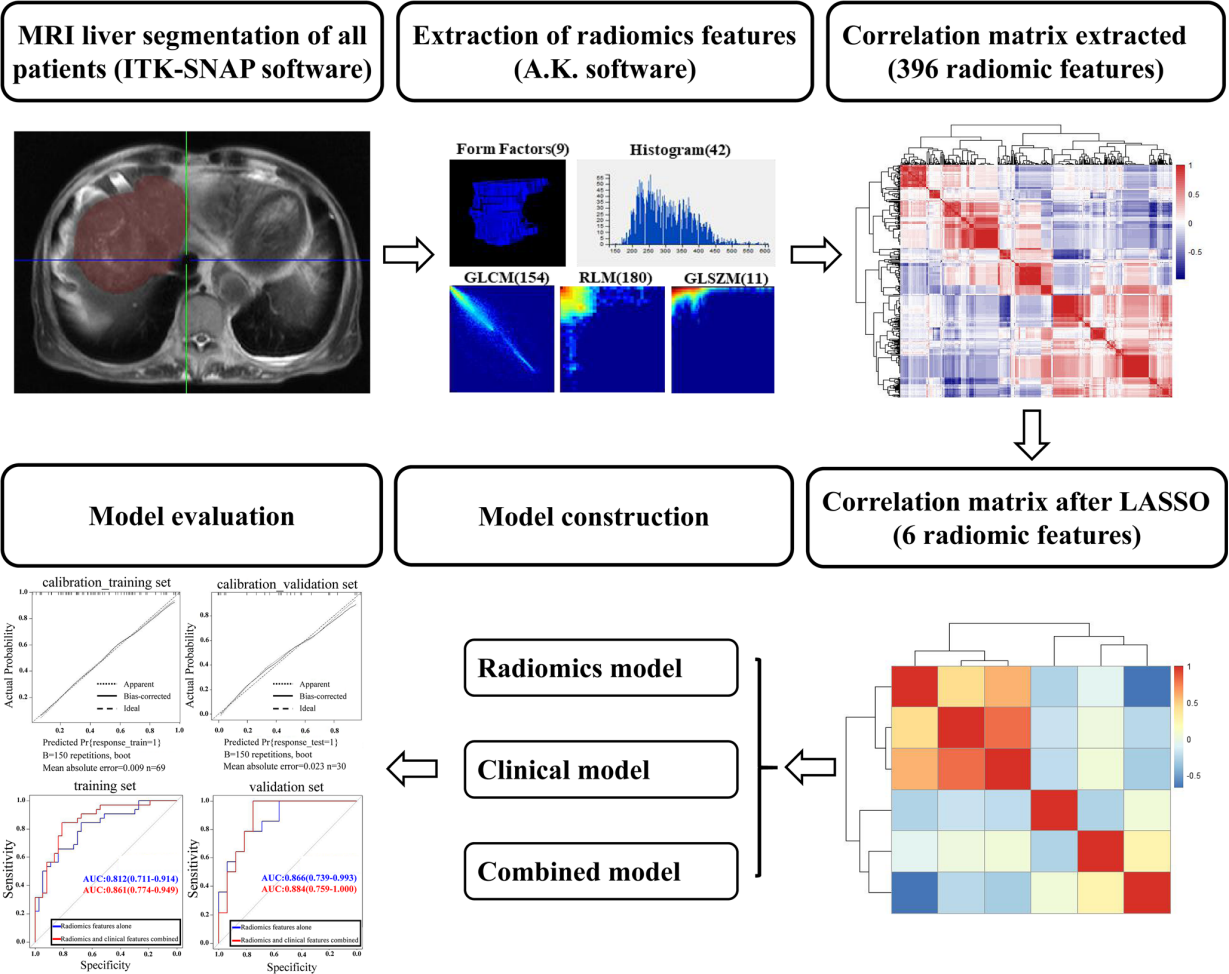
Flowchart of the study. The flowchart shows the overall operational process. First, VOIs were manually depicted based on raw MR imaging with ITK-SNAP software. Second, 396 features were extracted from the VOI of each patient by using the A.K. software, which had high correlation, as shown by the correlation matrix. Third, the LASSO method was applied for data dimension reduction, feature selection, and model construction. Finally, the performance of the radiomics features model, clinical features model, and the combined model were evaluated by the AUCs of the ROC curves, calibration curves, and decision curves. VOI, volume of interest; MR, magnetic resonance; LASSO, least absolute shrinkage and selection operator; AUC, area under the curve; ROC, receiver operating characteristic

**Fig. 4:**
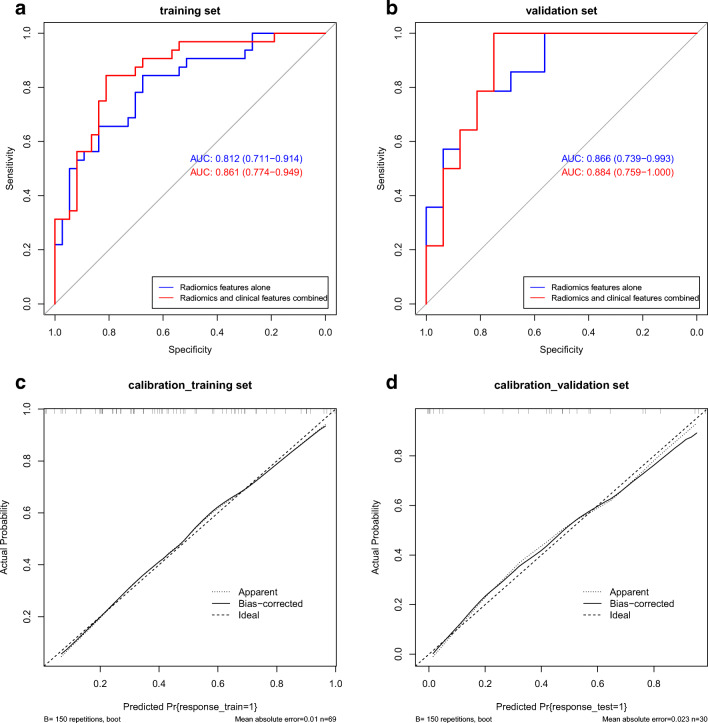
The predictive performance of each of the selected features and the Rad-score-based model. **a**, **b** ROC curves of each of the selected imaging features in the training and validation cohorts, respectively. **c**, **d** The predictive performance of the Rad-score-based model, as presented by ROC curves in the training cohort (AUC = 0.812, 95% CI: 0.711 to 0.914) and validation cohort (AUC = 0.866, 95% CI: 0.739 to 0.993). ROC, receiver operating characteristic; AUC, area under the curve; 95% CI, 95% confidence interval

The workflow of radiomics in this study is shown in Fig. 3 and Figure S1. Ultimately, six features that were most strongly associated with the TR were selected by LASSO logistic regression: variance, InverseDifferenceMoment_angle90_offset7, LongRunEmphasis_angle90_offset7, ShortRunEmphasis_AllDirection_offset4_SD, ShortRunEmphasis_angle135_offset1, and HighIntensityLargeAreaEmphasis (Table 2 and Figure S1C). The ROC curves for the selected features are presented in Fig. 4a and b, and the corresponding AUC, sensitivity, and specificity are shown in Table 2.

**Table 2:** Predictive performance of each feature selected by LASSO in the training and validation cohorts

Category	Feature selected	Coefficient	AUC		Sensitivity		Specificity	
			Training	Validation	Training	Validation	Training	Validation
GLSZM	Variance	−0.4574	0.613	0.728	93.8%	57.1%	29.7%	93.8%
GLCM	InverseDifferenceMoment_angle90_offset7	0.3763	0.610	0.522	37.5%	50.0%	83.8%	75.0%
RLM	LongRunEmphasis_angle90_offset7	0.9905	0.761	0.688	65.6%	100%	81.1%	43.8%
RLM	ShortRunEmphasis_AllDirection_offset4_SD	−0.9054	0.612	0.661	43.8%	42.9%	81.1%	100%
RLM	ShortRunEmphasis_angle135_offset1	−0.4763	0.694	0.701	62.5%	92.9%	73.0%	56.2%
Histogram	HighIntensityLargeAreaEmphasis	−0.0684	0.687	0.812	53.1%	64.3%	78.4%	93.8%
	Constant	−0.1068						

*LASSO*, least absolute shrinkage and selection operator; *AUC*, the area under the operating characteristic curve; *GLSZM*, gray-level size zone matrix; *GLCM*, gray-level co-occurrence matrix; *RLM*, run-length matrix

Then, all six selected features were combined to generate the Rad-score for each case to construct a prediction model for TR. The model showed excellent predictive performance in the training and validation cohorts (Fig. 4c, d). In the training cohort, the AUC of the prediction model was 0.812 (95% CI: 0.711 to 0.914), and the specificity and sensitivity were 0.676 and 0.844, respectively. In the validation cohort, the AUC, specificity, and sensitivity were 0.866 (95% CI: 0.739 to 0.993), 0.812, and 0.786, respectively.

**Fig. 5:**
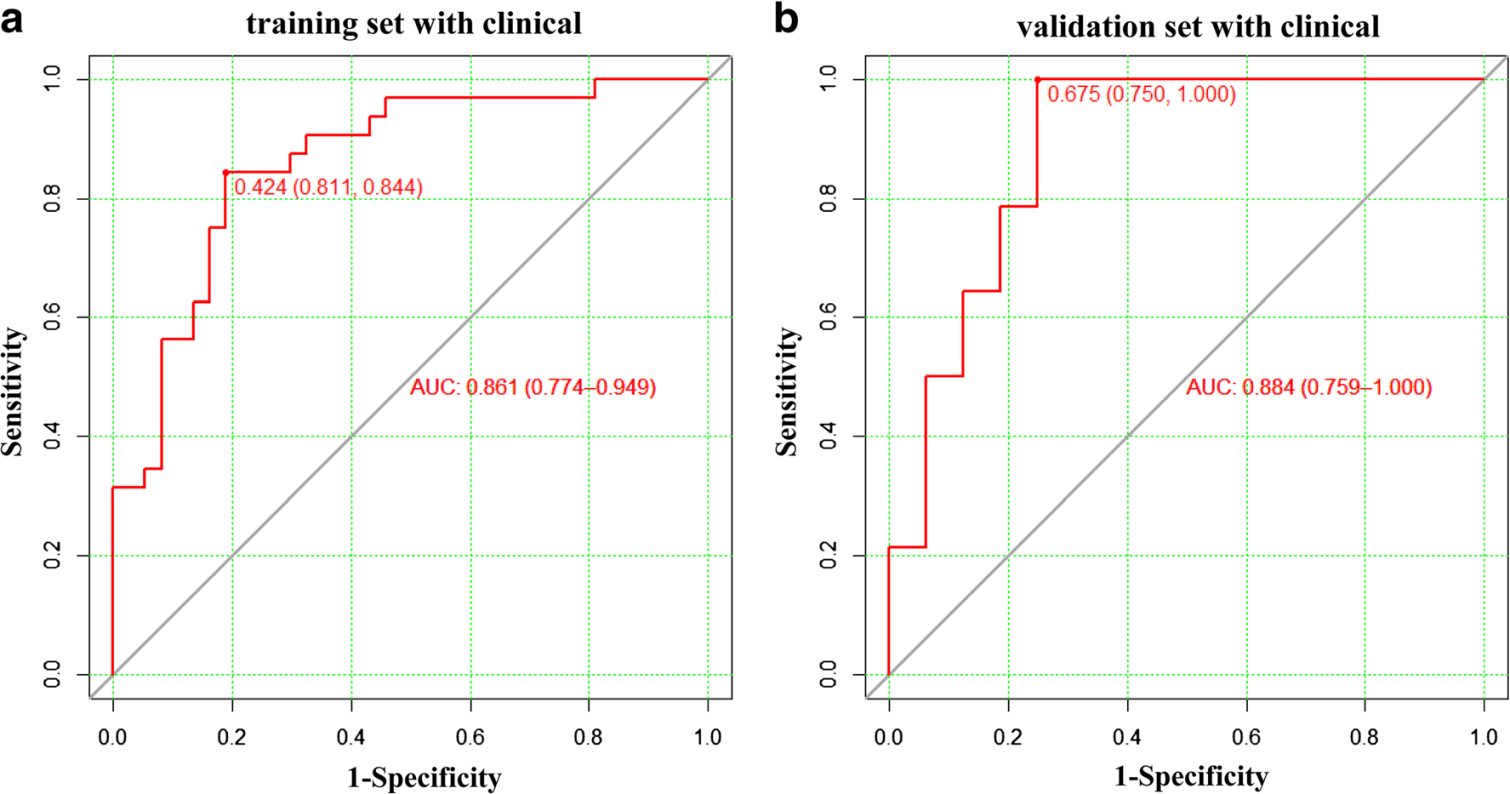
The predictive performance of the model based on the Rad-score and clinical indicators. **a**, **b** ROC curves of the combined model in the training and validation cohorts, respectively. ROC, receiver operating characteristic

To improve the prediction efficiency of the above model based on the Rad-score, we further integrated the screened clinical indicators (Child-Pugh class, BCLC stage, and AFP level) into the prediction model to form a novel model, and the performance of the model was improved significantly (Fig. 5 and Table 3). The AUC of the training cohort was 0.861 (95% CI: 0.774 to 0.949), and the specificity and sensitivity were as high as 0.811 and 0.844, respectively. The AUC of the validation cohort was 0.884 (95% CI: 0.764 to 1.000), and the specificity and sensitivity were 0.75 and 1.00, respectively.

**Table 3:** Comparison of the predictive performance for tumor response of each model in the training and validation cohorts

Models	Training cohort			Validation cohort		
	AUC	Sensitivity	Specificity	AUC	Sensitivity	Specificity
Radiomics model	0.812	84.4%	67.6%	0.866	78.6%	81.2%
Clinical model	0.733	62.5%	73.0%	0.667	42.9%	93.8%
Combined model	0.861	84.4%	81.1%	0.884	100%	68.8%

*AUC*, the area under the operating characteristic curve

### Construction of the predictive nomogram

**Fig. 6:**
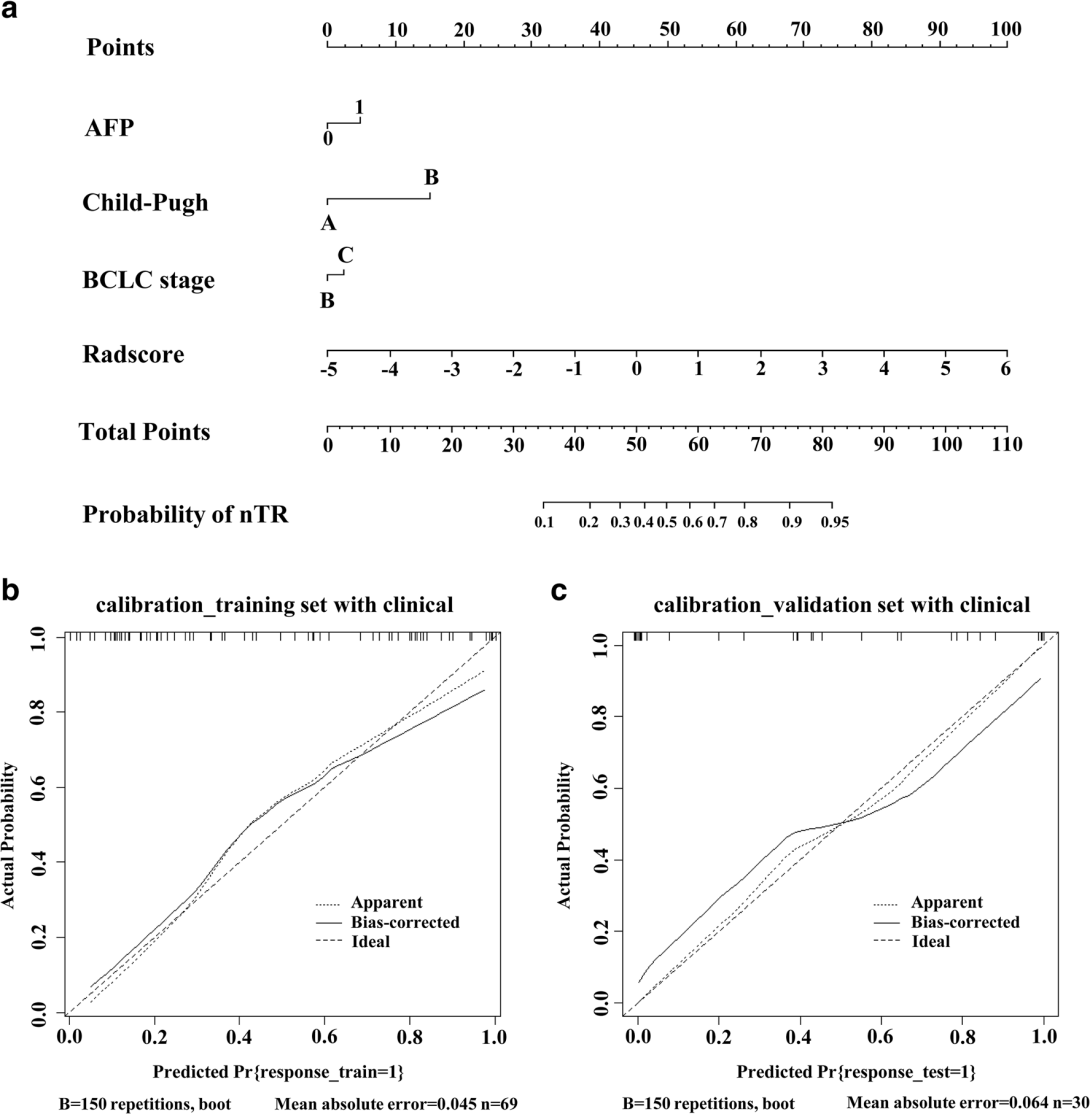
The nomogram and calibration curves of the model based on the radiomics signature and clinical predictors. **a** The radiomics nomogram integrated the radiomics signature with the AFP level, Child-Pugh class, and BCLC stage in the training cohort. The probability value of each HCC patient with nTR is marked on each axis. **b**, **c** The calibration curves between the nomogram prediction and actual observation for nTR and TR patients in the training and validation cohorts, respectively. The diagonal dotted line represents an ideal evaluation, while the solid lines and dashed lines represent the performance of the corrected and apparent bias, respectively. The closer the fit is to the diagonal dotted line, the better the evaluation. AFP, alpha-fetoprotein; BCLC, Barcelona Clinic Liver Cancer; HCC, hepatocellular carcinoma; TACE, transcatheter arterial chemoembolization; TR, TACE response; nTR, non-TACE response

A radiomics nomogram was constructed on the basis of the above novel prediction model, which effectively incorporated both the Rad-score and selected clinical predictors (Fig. 6a). The calibration plot also indicated good agreement between the nomogram prediction and actual observation for patients in both the training and validation cohorts (Fig. 6b, c).

### Clinical utility of the predictive nomogram

The decision curve analysis for the radiomics nomogram is presented in Figure S2. The decision curve showed that if the threshold probability of a patient was > 7%, using the radiomics nomogram to predict nTR adds more benefit than either the treat-none scheme or the treat-all-patients scheme. Within this range, on the basis of the above radiomics nomogram, the net benefit was comparable, with several overlaps.

## Discussion

TACE is a widely used locoregional palliative interventional therapy, and it plays a pivotal role in the management of HCC patients due to the blockage of the blood-supplying artery of tumors, which could cause extensive necrosis and inhibit tumor progression [23]. However, the clinical response of HCC patients to TACE tends to present large individual differences [8, 24]. Therefore, it is particularly important to accurately assess the tumor response before TACE, which is also essential for the further management of HCC. The present study developed and validated a novel predictive, radiomics signature and clinical predictor-based radiomics nomogram for predicting the responsiveness of TACE candidates with HCC. The model could successfully stratify patients into nTR and TR patients according to their pre-TACE Rad-scores and clinical characteristics.

In the present study, radiomics technology was applied to extract texture features in T2WI of HCC patients, and 396 candidate radiomics features were applied for further study. Among these 396 radiomics features, there are 42 histogram-based features, 9 form factor-based features, 11 GLSZM-based features, 180 RLM-based features, and 154 GLCM-based features. The histogram-based features were calculated using the intensity (HU) distribution of a given ROI and reflect the intensity information of tumors [25]. The form factor-based parameters can describe the three-dimensional size and shape of the tumor region. The GLSZM-based features compute the number of times a pixel with a specific gray-level occurs with another pixel with a gray value jointly. It represents the joint probability of specific pixels having certain gray-level values [26]. The RLM-based features were defined as the number of runs with pixels of different gray levels and run lengths for a given direction such as those defined by GLCM [27].

In our study, the potential 396 candidate radiomics features were finally reduced to six potential predictors by shrinking the regression coefficients with the LASSO method for further integration to form the Rad-score, which contains effective biological information and could reflect the heterogeneity of the tumor [18]. Several previous studies have demonstrated that the Rad-score could effectively predict the prognosis of patients due to its high correlation with tumor biological characteristics [28, 29]. For TACE response, a recent study showed that the computed tomography–based radiomics signature was closely associated with the early recurrence of HCC after TACE [30]. These preliminary studies have further confirmed that the texture feature–based radiomics method is feasible for predicting TACE responsiveness.

The BCLC staging system and Child-Pugh class have been endorsed and recommended by multiple guidelines for prognosis and treatment stratification of HCC patients [31, 32], and plenty of studies have also demonstrated that BCLC stage and Child-Pugh class could be used as important indicators for the prognosis of HCC [33–35]. In addition, some studies have focused on the relationship between laboratory indicators (such as AFP, CEA, or HBsAg) and the prognosis of HCC [36–38]. In our study, univariate and multivariate analyses were also used to select the clinical predictors. The obtained results showed that BCLC stage, Child-Pugh class, and AFP level were highly correlated with TR and could be used as independent predictors. Then, the above three clinical predictors were combined with the radiomics signature to construct a novel TR prediction nomogram, which could effectively improve the predictive performance of the model based on the Rad-score.

The constructed novel TR prediction nomogram was further evaluated by a decision curve to clarify the clinical utility, which could offer insight into clinical outcomes on the basis of threshold probability, from which the net benefit could be derived (net benefit is defined as the differential value of true positives proportion and false positives proportion, weighted by the relative harm of false positive and false negative results) [39, 40]. The results showed that if the threshold probability of a patient is > 7%, using the radiomics nomogram in this study to predict poor tumor response of patients treated with TACE adds more benefit than either the treat-all-patients scheme or the treat-none scheme. The present novel nomogram provides an important quantitative indicator and reference for the decision-making and management of treatment regimens for clinical HCC patients.

The GIDEON study showed that cTACE and DEB-TACE comprise 74% and 16% of TACE procedures, respectively [41]. In clinic, the physicians chose cTACE or DEB-TACE for the treatment of HCC patients based upon the liver function of the patients, the number and size of tumors, whether discrete or diffuse, and their location in the livers. In order to obtain a universally applicable prognostic model for TACE, patients receiving cTACE or DEB-TACE were all enrolled in this study. The results showed that the TACE techniques were not an independent predictor for tumor response. The result is consistent with a recent study, which shows that there has been no statistical difference in efficacy (tumor response and overall survival) between cTACE and DEB-TACE [42]. Doxorubicin, epirubicin, and pirubicin are the most commonly used anticancer drugs in TACE treatment. A randomized controlled trial showed that there is no significant difference in survival among doxorubicin, cisplatin, and epirubicin [43]. The choice of the anticancer agent and the administered dose are recommended by the interventional radiologists based on the condition of the patients [44].

However, there are several limitations in our study. First, selection bias was inevitable because the current study was a single-center, retrospective study. Second, the sample size was relatively small, and external validation and larger datasets are needed to validate and refine our results. In the future, large samples, multi-center cohorts, and multimodal research should be conducted to validate the prognostic significance of this radiomics signature.

In conclusion, radiomics provides a novel method to extract potentially important data from clinical imaging that can identify different clusters. The constructed novel TR prediction nomogram is a novel, noninvasive, efficient, and feasible method for the prediction of clinical TACE responsiveness in HCC patients. The quantitative nomogram prediction model based on the Rad-score and clinical predictors may serve as an alternative method for precision medicine and provide highly informative data for making clinical management decisions. Furthermore, the present study provides new strategies for more precise and personalized management of HCC patients, which could also be extended to other types of treatment.

## Supplementary Information

ESM 1 (DOCX 246 kb)

## Abbreviations

95% CI: 95% confidence interval
BCLC: Barcelona Clinic Liver Cancer
GLCM: Gray-level co-occurrence matrix
GLSZM: Gray-level size zone matrix
LASSO: Least absolute shrinkage and selection operator
nTR: Non-TACE response
RLM: Run-length matrix
TR: TACE response

## Declarations

### Guarantor

The scientific guarantor of this publication is Jiansong Ji.

### Statistics and biometry

No complex statistical methods were necessary for this paper.

### Methodology

• retrospective

• diagnostic or prognostic study

• performed at one institution
