# Supplementary material for: Prediction of tumor response via a pretreatment MRI radiomics-based nomogram in HCC treated with TACE
Source: Eur Radiol. 2021 Apr 16;31(10):7500–11. doi: 10.1007/s00330-021-07910-0 (PMC8452577; doi:10.1007/s00330-021-07910-0)
Supplement: Supplementary file 1 — (DOCX 246 kb) [file 330_2021_7910_MOESM1_ESM.docx]

**Supplementary material**

**Figure S1.** Feature selection and dimension reduction. (A) The 10-fold cross-validation of the LASSO analysis was applied to acquire the most valuable features. (B) The regression coefficients of LASSO. (C) The importance of the 6 features.

**Figure S2.** Decision curve analysis for the radiomics nomogram and the model with addition of clinical predictors. The y-axis measures the net beneﬁt. The red line represents the radiomics nomogram. The green line represents the model with addition of clinical predictors. The blue line represents the assumption that all patients have poor prognosis. The orange line represents the assumption that all patients are of good prognosis. The decision curve showed that if the threshold probability of a patient is >7%, using the radiomics nomogram in the current study to predict poor prognosis of patients treated with TACE adds more benefit than either the treat-all-patients scheme or the treat-none scheme.

**Figure S1**

**
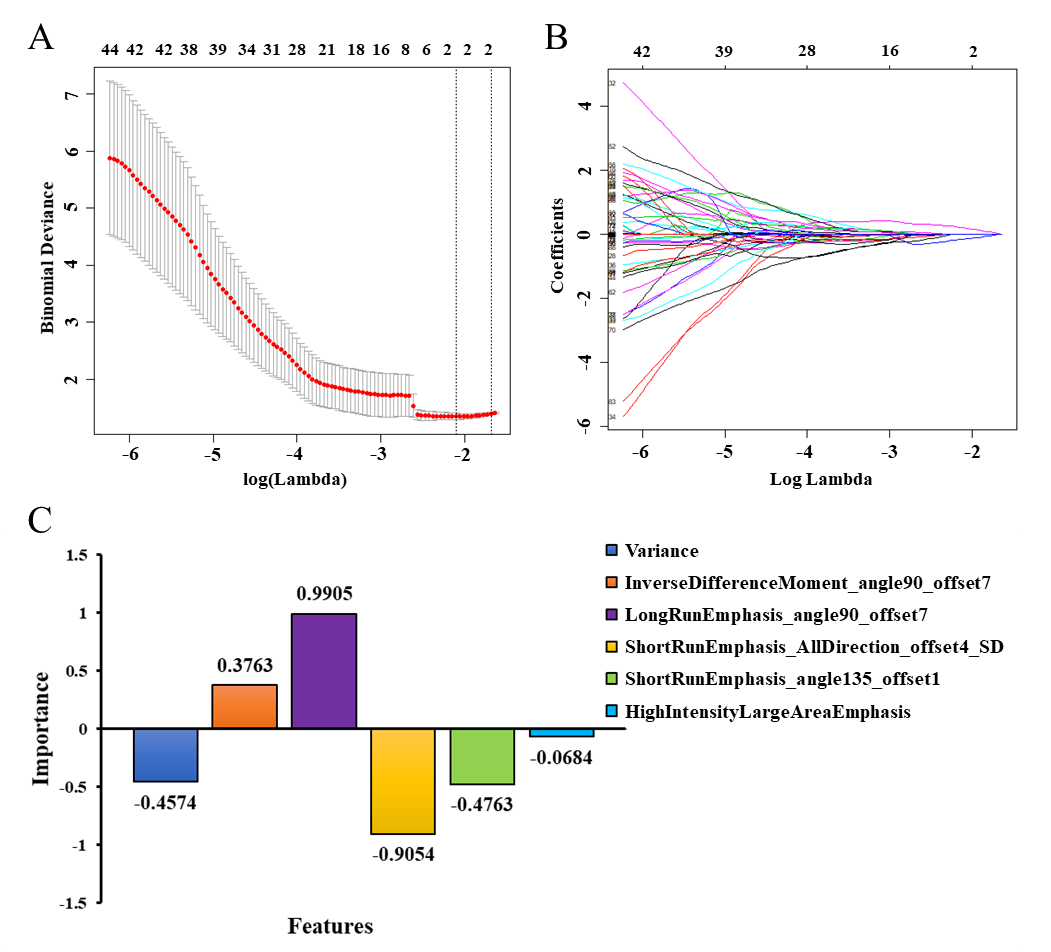
**

**Figure S2**

**
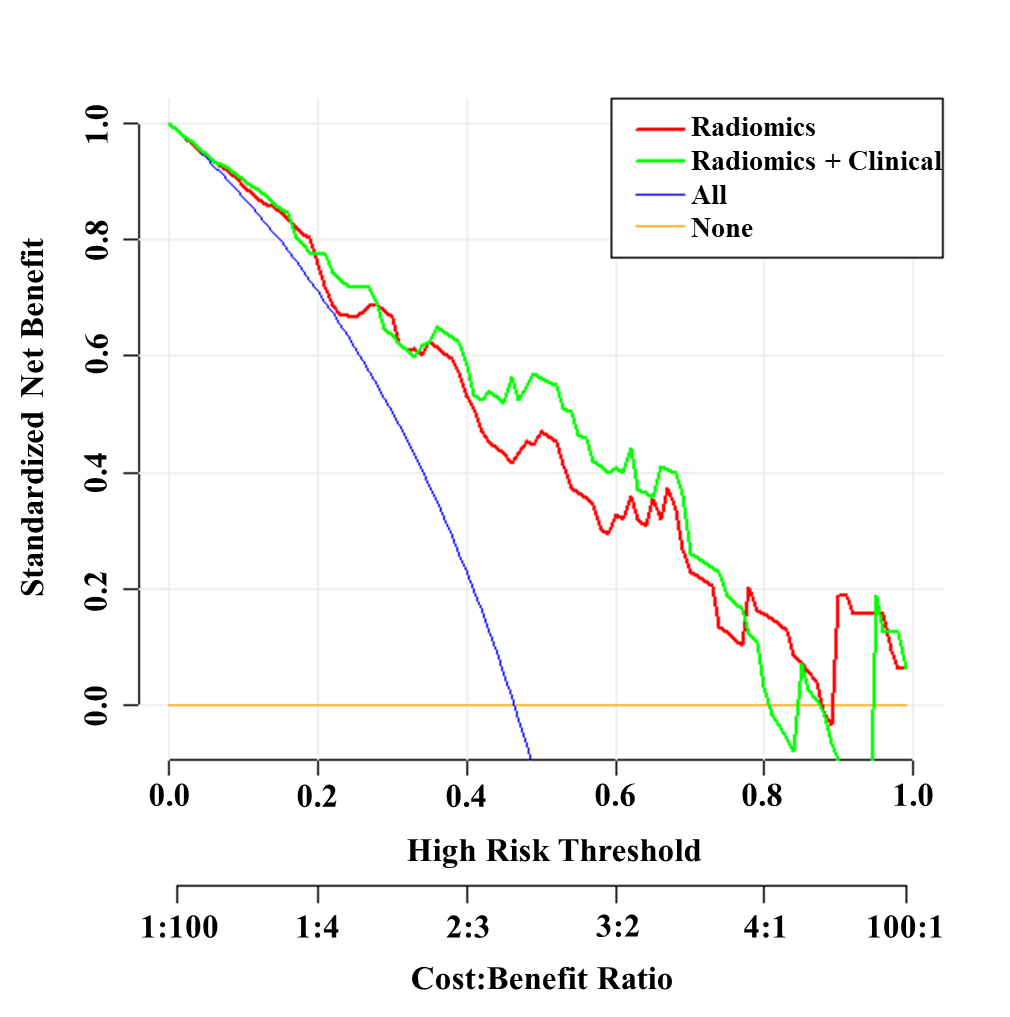
**
